# Hematologic Neoplasms Associated with Down Syndrome: Cellular and Molecular Heterogeneity of the Diseases

**DOI:** 10.3390/ijms242015325

**Published:** 2023-10-18

**Authors:** Edoardo Peroni, Michele Gottardi, Lucia D’Antona, Maria Luigia Randi, Antonio Rosato, Giacomo Coltro

**Affiliations:** 1 Immunology and Molecular Oncology Unit, Veneto Institute of Oncology, IOV-IRCCS, 35128 Padova, Italy; 2 Onco Hematology, Department of Oncology, Veneto Institute of Oncology, IOV-IRCCS, 31033 Padua, Italy; 3 Medical Genetics Unit, Mater Domini University Hospital, 88100 Catanzaro, Italy; 4 First Medical Clinic, Department of Medicine-DIMED, University of Padova, 35128 Padova, Italy; 5 Department of Surgery Oncology and Gastroenterology, University of Padova, 35122 Padova, Italy; 6 Department of Clinical and Experimental Medicine, University of Florence, 50134 Florence, Italy; 7 Center of Research and Innovation for Myeloproliferative Neoplasms, CRIMM, AOU Careggi, 50134 Florence, Italy

**Keywords:** acute myeloid leukemia, acute lymphoblastic leukemia, single-cell RNA sequencing, Down syndrome, trisomy 21, personalized medicine, multi-omics approach

## Abstract

The molecular basis of Down syndrome (DS) predisposition to leukemia is not fully understood but involves various factors such as chromosomal abnormalities, oncogenic mutations, epigenetic alterations, and changes in selection dynamics. Myeloid leukemia associated with DS (ML-DS) is preceded by a preleukemic phase called transient abnormal myelopoiesis driven by *GATA1* gene mutations and progresses to ML-DS via additional mutations in cohesin genes, *CTCF*, *RAS*, or *JAK/STAT* pathway genes. DS-related ALL (ALL-DS) differs from non-DS ALL in terms of cytogenetic subgroups and genetic driver events, and the aberrant expression of *CRLF2*, *JAK2* mutations, and *RAS* pathway-activating mutations are frequent in ALL-DS. Recent advancements in single-cell multi-omics technologies have provided unprecedented insights into the cellular and molecular heterogeneity of DS-associated hematologic neoplasms. Single-cell RNA sequencing and digital spatial profiling enable the identification of rare cell subpopulations, characterization of clonal evolution dynamics, and exploration of the tumor microenvironment’s role. These approaches may help identify new druggable targets and tailor therapeutic interventions based on distinct molecular profiles, ultimately improving patient outcomes with the potential to guide personalized medicine approaches and the development of targeted therapies.

## 1. Introduction

### Trisomy 21 and Leukemia

Down syndrome (DS) is the most common chromosomal disorder in humans, with a prevalence of approximately 1/700 live births worldwide [1]. It results from a full trisomy of chromosome 21 (T21) in 90% of cases, with the remaining patients harboring other chromosome 21 abnormalities or mosaicisms [2]. Of note, while the risk of solid tumors is reduced throughout life, DS is associated with an increased risk of developing leukemia, especially during the first years of life [3,4,5]. In particular, DS children have a 150-fold and 7- to 20-fold increased risk of developing acute myeloid leukemia (AML) and acute lymphoblastic leukemia (ALL), respectively, compared to non-DS individuals [3,6]. Accordingly, the 2022 International Consensus Classification includes myeloid/lymphoid neoplasms associated with DS among the category of “Hematologic neoplasms with germline predisposition associated with a constitutional disorder affecting multiple organ systems” [7]. Although ALL is much more common in the general pediatric population than in AML, with a ratio of 6.5, DS decreases this ratio to 1.7 [3].

Despite recent scientific progress, the molecular basis of DS predisposition to leukemia remains elusive. Patently, the perturbation of hematopoiesis in DS individuals is driven by chromosome 21, encoding >200 genes with direct and indirect effects on cellular homeostasis. This is further reinforced by the observation that the gain of chromosome 21 is one of the most frequent cytogenetic abnormalities across all subtypes of hematological malignancies, whereas it is rarely seen in solid tumors [8,9]. Besides T21-related mechanisms, several additional drivers have been described to be involved in DS-associated leukemogenesis, including concomitant oncogenic mutations, epigenetic and transcriptional alterations, and changes in selection dynamics within the fetal liver niche [10]. Recently, single-cell multi-omics technologies have led to unbiased investigation of cellular profiles at unprecedented resolution in all hematological areas, including DS-associated hematologic neoplasms [11,12].

Overall, DS represents the human phenotype model of genomic gain dosage imbalances [1], and the implementation of emerging single-cell analyses constitutes an unprecedented opportunity to decipher the molecular consequences of genome dosage imbalance with potential groundbreaking consequences for non-DS leukemogenesis as well.

## 2. Result and Discussion

### 2.1. Myeloid Proliferations Related to DS

These constitute a unique model of stepwise leukemogenesis that occurs during both pre- and post-natal life. Myeloid leukemia associated with DS (ML-DS) generally, although not always, phenotypically recapitulates the features of acute megakaryoblastic leukemia (AMKL). Virtually all cases of ML-DS are preceded by a preleukemic phase termed transient abnormal myelopoiesis (TAM), which occurs in 5–30% of all DS neonates and is characterized based on the clonal proliferation of immature myeloid cells (mostly megakaryoblasts) driven by somatic mutations in the gene encoding for the erythroid-megakaryocyte (MK) transcription factor GATA binding protein 1 (*GATA1*) [13,14,15]. Mutations in *GATA1* arise in utero from the 21st week of gestation, mostly located in exon 2, and result in the expression of a shorter N-terminal deleted protein (GATA1s) [13,14,15,16,17]. Although TAM spontaneously resolves within the first 3 months after birth in the majority of newborns, 20% of patients subsequently develop ML-DS before the age of five years [18]. Leukemic progression occurs from the initial *GATA1*-mutated clone via the acquisition of additional mutations, predominantly loss-of-function, in genes of the cohesin complex, CCCTC-binding factor (*CTCF*), or mutations leading to constitutive activation of the RAS or JAK/STAT pathways [19,20,21]. However, it is essential to remember that a specific cause of fetal death in DS fetuses is the development of lethal AMKL in utero, which compromises blood vessels and organs; unfortunately, some of these cases are not diagnosed in stillborns if an autopsy or histological examination of the placenta is lost in the case series [22,23].

Unlike non-DS AMKL, ML-DS generally harbors a favorable prognosis with excellent overall survival (OS) [24,25,26]. Notwithstanding, outcomes are dismal in patients with refractory/relapsed disease, with an OS rate of <20% [26,27]. Discernibly, the prevention of leukemic development by targeting preleukemic clones represents an attractive therapeutic strategy.

In a recent paper, whole-genome sequencing (WGS) of clonally expanded single fetal cells showed that the mutation rate in hematopoietic stem and progenitor cells (HSPCs) is higher during fetal development compared to the post-infant rate life, and even further increased in DS in comparison to karyotypically normal fetuses [28]. Of interest, fetal T21 HSPCs displayed similar mutation loads and patterns as DS-TAM, suggesting that mutational processes during normal fetal hematopoiesis may account for clonal evolution in DS-associated myeloid preleukemia.

Using a multi-omics assessment of mRNA and multiplexed protein epitope expression, Jardine and colleagues [11] uncovered an intrinsic bias of fetal HSPCs in DS that is underpinned by genome-wide transcriptional changes. In detail, MKs from DS fetuses expressed higher levels of regulons for GABPA (encoded on chromosome 21) and lower levels of *FLI1* (a driver of MK differentiation), in line with previous data. Most myeloid lineages overexpressed *TNF* and TNF signaling pathway genes, which is consistent with the higher levels of circulating TNF in DS. Moreover, the expression of *NOTCH1* and NOTCH ligands *NOV* (*CCN3*) and *DLK1* was significantly higher in the endothelium and HSC/MPPs from fetuses with DS, respectively. These findings provide novel insights into the role of cell-intrinsic and cell-extrinsic regulation of differentiation in the development of ML-DS.

Recently, Wagenblast and colleagues [29] developed a humanized model that faithfully recapitulates the full spectrum of DS premalignant and malignant leukemia using CRISPR/Cas9-mediated gene editing of primary human disomic and trisomic fetal HSPCs followed by xenotransplantation. In detail, the introduction of *GATA1* mutations caused preleukemia only in long-term trisomic HSCs. The simultaneous overexpression of a subset of chromosome 21 microRNAs (*miR-99a*, *miR-125b-2*, and *miR-155*) contributed to preleukemia initiation, whereas their removal in T21 long-term HSCs inhibited GATA1s-induced preleukemia development. Furthermore, leukemic progression was independent of T21 and occurred in multiple HSPCs through additional mutations in the cohesin genes (including *STAG2*). Overall, these findings highlight the relevance of the cellular and developmental status of the cell of origin during leukemogenesis, reinforcing the idea that genetic drivers can be distinct between pediatric and adult AML. Interestingly, CD117+/KIT was identified as a driver of the propagation of preleukemia and leukemia cells, and pharmacological KIT inhibition targeted both GATA1-induced preleukemic and primary DS preleukemia patient cells.

To investigate the cooperation between GATA1s and secondary genetic abnormalities, Arkoun and colleagues used CRISPR/Cas9 technology to sequentially introduce *GATA1s*, *MPL*^W515K^, and haploinsufficiency of *SMC3* (cohesin complex subunit) in human disomic- and trisomic-induced pluripotent stem cells (iPSCs). GATA1s profoundly reshaped iPSC-derived hematopoiesis and cooperated with *SMC3* haploinsufficiency to induce an even more profound failure of the GATA1-dependent MK differentiation program, including *NFE2* downregulation. While T21 enhanced the proliferative phenotype, the impairment of MK differentiation was independent of T21, which is consistent with previous findings [29], thereby suggesting that leukemic progression is independent of T21 and can be induced by the synergistic interaction between GATA1s and cohesin gene mutations. 

### 2.2. Acute Lymphoblastic Leukemia Related to DS

Acute Lymphoblastic Leukemia associated with DS (ALL-DS) is a distinct entity with clinical and biological features that differ from those of non-DS ALL. The age of diagnosis extends into adolescence and young adulthood, with a peak age that is slightly higher compared to that of non-DS ALL [30]. The increased risk of ALL in DS is almost exclusively limited to the B-cell precursor (BCP) phenotype, with a few reported cases of T-cell ALL [30,31].

Likewise, the common cytogenetic subgroups of childhood non-DS-ALL are less represented in DS patients, including both favorable (i.e., *ETV6*-*RUNX1* and hyperdiploidy) and unfavorable (i.e., *BCR*-*ABL* and *MLL*-*AF4*) abnormalities [32,33]. Among the genetic driver events implicated in ALL-DS development, aberrant expression of *CRLF2* (cytokine receptor-like factor 2) has been well characterized. CRLF2 is an important lymphoid signaling receptor, and its dysregulation is found in 5–15% of sporadic B-ALL cases versus 60% of ALL-DS [33,34]. Other studies uncovered a high frequency of *JAK2* mutations in ALL-DS, particularly the R683 mutation, which can be found in 18–28% of patients [35,36,37]. Interestingly, several studies reported a tight association between aberrant *CRLF2* expression and *JAK2* mutations, suggesting cooperation between the two alterations [34,38]. In addition to and mutually exclusively with *JAK* mutations, some reports identified RAS pathway-activating mutations in up to 30% of ALL-DS patients [39,40].

Compared to ML-DS and other childhood malignancies, ALL-DS has so far remained poorly characterized. In a study investigating clonal evolution in 47 patients with *CRLF2*-rearranged B-ALL, *CRLF2* abnormalities were found to be early events in DS(*12*). Moreover, DS patients showed a complex branching tree structure with multiple sub-clonal events, in contrast to non-DS patients, who appeared to evolve in a linear non-branching manner.

In the attempt to investigate the mechanisms underlying clonal selection in childhood BCP-ALL during induction chemotherapy, Turati and colleagues [41] used high-depth WGS followed by single-cell qPCR on multiple patient-derived xenograft recipients after treatment with vincristine and dexamethasone. They found that chemotherapy, while having little impact on genetic heterogeneity, exerts an extensive action on the transcriptional and epigenetic profiles, resulting in a bottleneck selection of a genetically polyclonal but phenotypically uniform population with hallmark signatures related to developmental stage, cell cycle, and metabolism.

In a more recent study [42], the same study group presented compelling data suggesting that specific genotype–phenotype relationships have functional relevance in terms of leukemic progression and treatment resistance. Using serial transplantation assays and SNP-arrays, the authors showed that individual genetic lesions are restricted to well-defined cell immunophenotypes, corresponding to different stages of the leukemic differentiation hierarchy. The reconstruction of the leukemia phylogenetic tree demonstrated that the dominant population at relapse originated from a rare, highly quiescent, and developmentally primitive clone, representing a reservoir for relapse. More interestingly, genotypes and phenotypes with functional relevance were shown to co-segregate within the disease, in contrast to previous findings in non-DS ALL [41]. Overall, these findings indicate that ALL-DS is a complex matrix of cells exhibiting extensive genetic and epigenetic heterogeneity with dynamic clonal evolution and competition and reinforce the idea that chemotherapy can act as a critical selective force for the preferential expansion of selected leukemic compartments and subsequent relapse.

In remarkable contrast to the excellent prognosis of ML-DS, patients with ALL-DS typically have poor outcomes, with lower OS (35.7% vs. 75.8%) and higher relapse rates (28.5% vs. 13.3%) compared to their non-DS counterparts [43]. Moreover, patients with ALL-DS have a higher susceptibility to chemotherapy-related toxicities, resulting in an increased risk of treatment-related morbidities and mortalities. On that basis, the treatment of ALL-DS patients can be challenging, and the identification of specific molecular features aimed at reducing both relapse risk and treatment-related toxicities represents a major goal for the future [44].

## 3. Material and Methods

### Single-Cell Analysis, Extending the Frontiers of ML/ALL-DS

In recent years, next-generation sequencing (NGS) significantly contributed to the understanding of cancer heterogeneity and subclonal evolution, mechanisms of treatment resistance and relapse, and identification of actionable mutations. Nevertheless, bulk genomic profiling methods fail to accurately resolve the clonal architecture of tumor populations and are limited to the identification of relapse-driving clones.

The development of single-cell analysis techniques, including single-cell RNA sequencing (scRNA-seq) and spatial single-cell imaging, offers a promising opportunity to gain insights into the disease. The use of scRNA-seq may allow the identification of rare cell subpopulations and the characterization of clonal evolution dynamics in order to decipher the mechanisms supporting treatment resistance and disease relapse [45]. Moreover, scRNA-seq and digital spatial profiling have the potential to investigate the role of tumor microenvironment (TME) in supporting leukemia cell growth and survival. Ultimately, such depth of exploration provided by single-cell approaches may allow for the development of novel targeted therapies aimed at improving the outcomes of patients with ML/ALL-DS.

**Figure 1:**
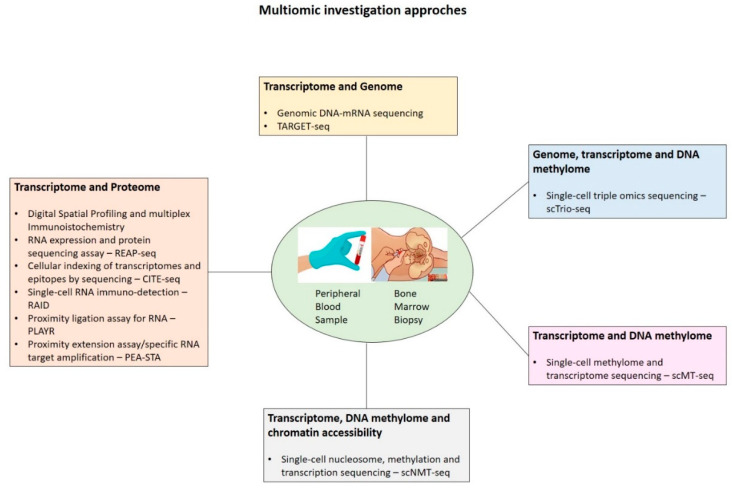
Several multi-omics approaches combining genomic, transcriptomic, proteomic, and spatial imaging data from bone marrow biopsy and/or peripheral blood samples.

Finally, the integration of single-cell techniques with multi-omics approaches (Figure 1) will be able to transform our understanding of the biology of DS leukemogenesis. The joint analysis of genome, transcriptome, epigenome, and proteome at the single-cell level may provide pivotal new insights into the complex interplay between intracellular and intercellular molecular mechanisms driving disease pathogenesis, evolution, and recurrence. Understanding the proteome and epigenome from chemical modifications to the DNA molecule itself to the histone proteins associated with DNA is crucial in comprehending the molecular mechanisms underlying DS-related leukemia. Studying the proteome provides insights into dysregulated cellular pathways in leukemia. Epigenetic modifications, such as DNA methylation and histone modifications, play a significant role in gene expression regulation. In DS-related leukemia, aberrant epigenetic modifications can lead to the activation or silencing of specific genes, thereby contributing to the development of leukemia. Understanding the epigenetic landscape of DS-related leukemia can lead to the development of therapies targeting these modifications. Epigenetic drugs, like DNA methyltransferase inhibitors and histone deacetylase inhibitors, can reverse abnormal epigenetic patterns, potentially restoring normal gene expression and slowing down the progression of the disease, such as guadecitabine, non-nucleoside DNMT inhibitors, DOTIL inhibitors, or BET inhibitors (Molibresib, GSK525762, INCB057643, ODM-207, RO6870810, BAY 1238097, FT-1101) [46].

## 4. Conclusions

Once again, all these technological innovations are ultimately being put to the service of precision medicine via the identification of more tailored and effective therapies for ML/ALL-DS.

In conclusion, DS leukemogenesis represents a unique disease setting to study human preleukemia and the evolutionary steps that lead to fully transformed leukemia. Recently, the increasingly widespread access to platforms based on cell-by-cell technologies has allowed overcoming the limitations of conventional bulk NGS analysis while shedding light on the complexity of tumor composition and clonal evolution. In the era of personalized medicine, single-cell research has the potential to provide a more detailed understanding of the biology of DS leukemogenesis in order to identify new druggable targets and tailor the therapeutic interventions according to distinct molecular profiles at risk.

## Data Availability

Not applicable.

## References

[1] Antonarakis S.E. (2017). Down syndrome and the complexity of genome dosage imbalance. Nat. Rev. Genet..

[2] Antonarakis S.E., Skotko B.G., Rafii M.S., Strydom A., Pape S.E., Bianchi D.W., Sherman S.L., Reeves R.H. (2020). Down syndrome. Nat. Rev. Dis. Primers.

[3] Hasle H., Clemmensen I.H., Mikkelsen M. (2000). Risks of leukaemia and solid tumours in individuals with Down’s syndrome. Lancet.

[4] Hasle H. (2001). Pattern of malignant disorders in individuals with Down’s syndrome. Lancet Oncol..

[5] Ross J.A., Spector L.G., Robison L.L., Olshan A.F. (2005). Epidemiology of leukemia in children with Down syndrome. Pediatr. Blood Cancer.

[6] Khan I., Malinge S., Crispino J. (2011). Myeloid leukemia in Down syndrome. Crit. Rev. Oncog..

[7] Arber D.A., Orazi A., Hasserjian R.P., Borowitz M.J., Calvo K.R., Kvasnicka H.-M., Wang S.A., Bagg A., Barbui T., Branford S. (2022). International Consensus Classification of Myeloid Neoplasms and Acute Leukemias: Integrating morphologic, clinical, and genomic data. Blood J. Am. Soc. Hematol..

[8] Mitelman F., Heim S., Mandahl N. (1990). Trisomy 21 in neoplastic cells. Am. J. Med. Genet..

[9] Laurent A.P., Kotecha R.S., Malinge S. (2020). Gain of chromosome 21 in hematological malignancies: Lessons from studying leukemia in children with Down syndrome. Leukemia.

[10] Hasaart K.A., Bertrums E.J., Manders F., Goemans B.F., van Boxtel R. (2021). Increased risk of leukaemia in children with Down syndrome: A somatic evolutionary view. Expert. Rev. Mol. Med..

[11] Jardine L., Webb S., Goh I., Quiroga Londoño M., Reynolds G., Mather M., Olabi B., Stephenson E., Botting R.A., Horsfall D. (2021). Blood and immune development in human fetal bone marrow and Down syndrome. Nature.

[12] Potter N., Jones L., Blair H., Strehl S., Harrison C., Greaves M., Kearney L., Russell L. (2019). Single-cell analysis identifies CRLF2 rearrangements as both early and late events in Down syndrome and non-Down syndrome acute lymphoblastic leukaemia. Leukemia.

[13] Wechsler J., Greene M., McDevitt M.A., Anastasi J., Karp J.E., Le Beau M.M., Crispino J.D. (2002). Acquired mutations in GATA1 in the megakaryoblastic leukemia of Down syndrome. Nat. Genet..

[14] Hitzler J.K., Cheung J., Li Y., Scherer S.W., Zipursky A. (2003). GATA1 mutations in transient leukemia and acute megakaryoblastic leukemia of Down syndrome. Blood.

[15] Roberts I., Alford K., Hall G., Juban G., Richmond H., Norton A., Vallance G., Perkins K., Marchi E., McGowan S. (2013). GATA1-mutant clones are frequent and often unsuspected in babies with Down syndrome: Identification of a population at risk of leukemia. Blood J. Am. Soc. Hematol..

[16] Taub J.W., Mundschau G., Ge Y., Poulik J.M., Qureshi F., Jensen T., James S.J., Matherly L.H., Wechsler J., Crispino J.D. (2004). Prenatal origin of GATA1 mutations may be an initiating step in the development of megakaryocytic leukemia in Down syndrome. Blood.

[17] Hoeller S., Bihl M.P., Tzankov A., Chaffard R., Hirschmann P., Miny P., Kühne T., Bruder E. (2014). Morphologic and GATA1 sequencing analysis of hematopoiesis in fetuses with trisomy 21. Hum. Pathol..

[18] Gamis A.S., Alonzo T.A., Gerbing R.B., Hilden J.M., Sorrell A.D., Sharma M., Loew T.W., Arceci R.J., Barnard D., Doyle J. (2011). Natural history of transient myeloproliferative disorder clinically diagnosed in Down syndrome neonates: A report from the Children’s Oncology Group Study A2971. Blood J. Am. Soc. Hematol..

[19] Li Z., Godinho F.J., Klusmann J.-H., Garriga-Canut M., Yu C., Orkin S.H. (2005). Developmental stage–selective effect of somatically mutated leukemogenic transcription factor GATA1. Nat. Genet..

[20] Labuhn M., Perkins K., Matzk S., Varghese L., Garnett C., Papaemmanuil E., Metzner M., Kennedy A., Amstislavskiy V., Risch T. (2019). Mechanisms of progression of myeloid preleukemia to transformed myeloid leukemia in children with Down syndrome. Cancer Cell.

[21] Yoshida K., Toki T., Okuno Y., Kanezaki R., Shiraishi Y., Sato-Otsubo A., Sanada M., Park M.-J., Terui K., Suzuki H. (2013). The landscape of somatic mutations in Down syndrome–related myeloid disorders. Nat. Genet..

[22] Federmann B., Fasan A., Kagan K.O., Haen S., Fend F. (2015). Transient abnormal myelopoiesis/acute megakaryoblastic leukemia diagnosed in the placenta of a stillborn Down syndrome fetus with targeted next-generation sequencing. Leukemia.

[23] Bonometti A., Lobascio G., Boveri E., Cesari S., Lecca M., Arossa A., Spinillo A., Errichiello E., Paulli M. (2021). Acute megakaryoblastic leukemia with a novel GATA1 mutation in a second trimester stillborn fetus with trisomy 21. Leuk. Lymphoma.

[24] Lange B.J., Kobrinsky N., Barnard D.R., Arthur D.C., Buckley J.D., Howells W.B., Gold S., Sanders J., Neudorf S., Smith F.O. (1998). Distinctive demography, biology, and outcome of acute myeloid leukemia and myelodysplastic syndrome in children with Down syndrome: Children’s Cancer Group Studies 2861 and 2891. Blood J. Am. Soc. Hematol..

[25] Qiao B., Austin A.A., Schymura M.J., Browne M.L. (2018). Characteristics and survival of children with acute leukemia with Down syndrome or other birth defects in New York State. Cancer Epidemiol..

[26] Gupte A., Al-Antary E.T., Edwards H., Ravindranath Y., Ge Y., Taub J.W. (2022). The paradox of Myeloid Leukemia associated with Down syndrome. Biochem. Pharmacol..

[27] Caldwell J.T., Ge Y., Taub J.W. (2014). Prognosis and management of acute myeloid leukemia in patients with Down syndrome. Expert. Rev. Hematol..

[28] Hasaart K.A., Manders F., van der Hoorn M.-L., Verheul M., Poplonski T., Kuijk E., de Sousa Lopes S.M.C., van Boxtel R. (2020). Mutation accumulation and developmental lineages in normal and Down syndrome human fetal haematopoiesis. Sci. Rep..

[29] Wagenblast E., Araújo J., Gan O.I., Cutting S.K., Murison A., Krivdova G., Azkanaz M., McLeod J.L., Smith S.A., Gratton B.A. (2021). Mapping the cellular origin and early evolution of leukemia in Down syndrome. Science.

[30] Izraeli S., Vora A., Zwaan C.M., Whitlock J. (2014). How I treat ALL in Down’s syndrome: Pathobiology and management. Blood J. Am. Soc. Hematol..

[31] Buitenkamp T.D., Izraeli S., Zimmermann M., Forestier E., Heerema N.A., van Den Heuvel-Eibrink M.M., Pieters R., Korbijn C.M., Silverman L.B., Schmiegelow K. (2014). Acute lymphoblastic leukemia in children with Down syndrome: A retrospective analysis from the Ponte di Legno study group. Blood J. Am. Soc. Hematol..

[32] Forestier E., Izraeli S., Beverloo B., Haas O., Pession A., Michalová K., Stark B., Harrison C.J., Teigler-Schlegel A., Johansson B. (2008). Cytogenetic features of acute lymphoblastic and myeloid leukemias in pediatric patients with Down syndrome: An iBFM-SG study. Blood J. Am. Soc. Hematol..

[33] Lee P., Bhansali R., Izraeli S., Hijiya N., Crispino J.D. (2016). The biology, pathogenesis and clinical aspects of acute lymphoblastic leukemia in children with Down syndrome. Leukemia.

[34] Hertzberg L., Vendramini E., Ganmore I., Cazzaniga G., Schmitz M., Chalker J., Shiloh R., Iacobucci I., Shochat C., Zeligson S. (2010). Down syndrome acute lymphoblastic leukemia, a highly heterogeneous disease in which aberrant expression of CRLF2 is associated with mutated JAK2: A report from the International BFM Study Group. Blood J. Am. Soc. Hematol..

[35] Bercovich D., Ganmore I., Scott L.M., Wainreb G., Birger Y., Elimelech A., Shochat C., Cazzaniga G., Biondi A., Basso G. (2008). Mutations of JAK2 in acute lymphoblastic leukaemias associated with Down’s syndrome. Lancet.

[36] Kearney L., Gonzalez De Castro D., Yeung J., Procter J., Horsley S.W., Eguchi-Ishimae M., Bateman C.M., Anderson K., Chaplin T., Young B.D. (2009). Specific JAK2 mutation (JAK2 R683) and multiple gene deletions in Down syndrome acute lymphoblastic leukemia. Blood J. Am. Soc. Hematol..

[37] Gaikwad A., Rye C.L., Devidas M., Heerema N.A., Carroll A.J., Izraeli S., Plon S.E., Basso G., Pession A., Rabin K.R. (2009). Prevalence and clinical correlates of JAK2 mutations in Down syndrome acute lymphoblastic leukaemia. Br. J. Haematol..

[38] Mullighan C.G., Collins-Underwood J.R., Phillips L.A., Loudin M.G., Liu W., Zhang J., Ma J., Coustan-Smith E., Harvey R.C., Willman C.L. (2009). Rearrangement of CRLF2 in B-progenitor–and Down syndrome–associated acute lymphoblastic leukemia. Nat. Genet..

[39] Nikolaev S.I., Garieri M., Santoni F., Falconnet E., Ribaux P., Guipponi M., Murray A., Groet J., Giarin E., Basso G. (2014). Frequent cases of RAS-mutated Down syndrome acute lymphoblastic leukaemia lack JAK2 mutations. Nat. Commun..

[40] Koschut D., Ray D., Li Z., Giarin E., Groet J., Alić I., Kham S.K.-Y., Chng W.J., Ariffin H., Weinstock D.M. (2021). RAS-protein activation but not mutation status is an outcome predictor and unifying therapeutic target for high-risk acute lymphoblastic leukemia. Oncogene.

[41] Turati V.A., Guerra-Assunção J.A., Potter N.E., Gupta R., Ecker S., Daneviciute A., Tarabichi M., Webster A.P., Ding C., May G. (2021). Chemotherapy induces canalization of cell state in childhood B-cell precursor acute lymphoblastic leukemia. Nat. Cancer.

[42] Lutz C., Turati V.A., Clifford R., Woll P.S., Stiehl T., Castor A., Clark S.A., Ferry H., Buckle V., Trumpp A. (2022). Complex genotype-phenotype relationships shape the response to treatment of Down Syndrome Childhood Acute Lymphoblastic Leukaemia. bioRxiv.

[43] Schmidt M.-P., Colita A., Ivanov A.-V., Coriu D., Miron I.-C. (2021). Outcomes of patients with Down syndrome and acute leukemia: A retrospective observational study. Medicine.

[44] Page E.C., Heatley S.L., Yeung D.T., Thomas P.Q., White D.L. (2018). Precision medicine approaches may be the future for CRLF2 rearranged Down Syndrome Acute Lymphoblastic Leukaemia patients. Cancer Lett..

[45] Peroni E., Randi M.L., Rosato A., Cagnin S. (2023). Acute myeloid leukemia: From NGS, through scRNA-seq, to CAR-T. dissect cancer heterogeneity and tailor the treatment. J. Exp. Clin. Cancer Res..

[46] Nepali K., Liou J.P. (2021). Recent developments in epigenetic cancer therapeutics: Clinical advancement and emerging trends. J. Biomed. Sci..

